# Tumor-derived biomarkers predict efficacy of B7H3 antibody-drug conjugate treatment in metastatic prostate cancer models

**DOI:** 10.1172/JCI162148

**Published:** 2023-11-15

**Authors:** Supreet Agarwal, Lei Fang, Kerry McGowen, JuanJuan Yin, Joel Bowman, Anson T. Ku, Aian Neil Alilin, Eva Corey, Martine P. Roudier, Lawrence D. True, Ruth Dumpit, Ilsa Coleman, John K. Lee, Peter S. Nelson, Brian J. Capaldo, Aida Mariani, Clare Hoover, Ilya S. Senatorov, Michael Beshiri, Adam G. Sowalsky, Elaine M. Hurt, Kathleen Kelly

**Affiliations:** 1 Laboratory of Genitourinary Cancer Pathogenesis, Center for Cancer Research, National Cancer Institute, NIH, Bethesda, Maryland, USA.; 2 Department of Urology and; 3 Department of Laboratory Medicine and Pathology, University of Washington, Seattle, Washington, USA.; 4 Divisions of Human Biology and Clinical Research, Fred Hutchinson Cancer Research Center, Seattle, Washington, USA.; 5 AstraZeneca, Gaithersburg, Maryland, USA.

**Keywords:** Cell Biology, Therapeutics, Drug therapy, Prostate cancer

## Abstract

Antibody-drug conjugates (ADCs) are a promising targeted cancer therapy; however, patient selection based solely on target antigen expression without consideration for cytotoxic payload vulnerabilities has plateaued clinical benefits. Biomarkers to capture patients who might benefit from specific ADCs have not been systematically determined for any cancer. We present a comprehensive therapeutic and biomarker analysis of a B7H3-ADC with pyrrolobenzodiazepine(PBD) payload in 26 treatment-resistant, metastatic prostate cancer (mPC) models. B7H3 is a tumor-specific surface protein widely expressed in mPC, and PBD is a DNA cross–linking agent. B7H3 expression was necessary but not sufficient for B7H3-PBD-ADC responsiveness. RB1 deficiency and/or replication stress, characteristics of poor prognosis, and conferred sensitivity were associated with complete tumor regression in both neuroendocrine (NEPC) and androgen receptor positive (ARPC) prostate cancer models, even with low B7H3 levels. Non-ARPC models, which are currently lacking efficacious treatment, demonstrated the highest replication stress and were most sensitive to treatment. In RB1 WT ARPC tumors, SLFN11 expression or select DNA repair mutations in SLFN11 nonexpressors governed response. Importantly, WT TP53 predicted nonresponsiveness (7 of 8 models). Overall, biomarker-focused selection of models led to high efficacy of in vivo treatment. These data enable a paradigm shift to biomarker-driven trial designs for maximizing clinical benefit of ADC therapies.

## Graphical abstract

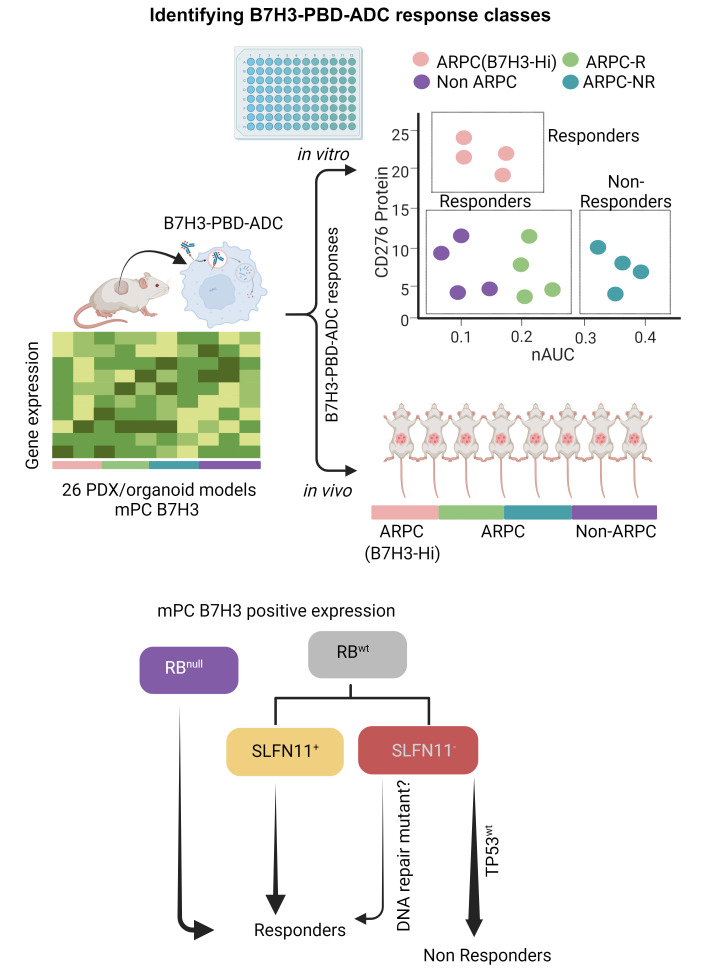

## Introduction

Metastatic prostate cancer (mPC) remains a lethal disease accounting for more than 30,000 deaths annually in the United States (1). The use of androgen deprivation therapy (ADT) and androgen receptor (AR) signaling inhibitors (ARSI) have substantially prolonged the survival of patients with mPC in both pre and postchemotherapy settings. Unfortunately, durable complete responses are uncommon and mortality rates approach 100% with the development of castration resistant (CR) metastatic prostate cancer (mCRPC). Continuously evolving acquired resistance mechanisms include frequent AR mutations and structural genomic alterations that drive an AR-positive prostate cancer (ARPC) adenocarcinoma phenotype. Less common, but increasing in frequency, are resistance mechanisms that bypass an AR requirement through lineage plasticity with the emergence of phenotypes spanning neuroendocrine prostate cancer (NEPC) phenotypes and various other histologies (2). Across the landscape of genomic alterations in mCRPC, retinoblastoma (RB) transcriptional corepressor 1 (*RB1)* alteration is the only genomic factor strongly associated with poor survival (3), highlighting the need for potential therapeutic strategies targeting RB1-deficient tumors. Since the vast majority of mPC phenotypes eventually resist all currently approved therapeutics, new treatment strategies are essential.

A promising approach for developing effective and less toxic therapies for mPC involves selectively targeting tumor cells via tumor-specific cell surface proteins and cognate antigens. Exploiting prostate specific membrane antigen (PSMA) to deliver high dose radiation, PSMA-Lu177, to tumor cells overexpressing PSMA has recently gained FDA approval for the treatment of mPC (4). PSMA is expressed by the majority of, though not all, ARPCs, but emerging treatment-resistant phenotypes such as AR-negative and small cell neuroendocrine PC (SCNPC) generally do not express PSMA, prompting a search for alternate targets. CD276/B7H3 is a type I transmembrane protein overexpressed in several solid tumors and often correlated with poor survival and higher tumor grade (5, 6). B7H3 is overexpressed in prostate cancer compared with benign prostatic hyperplasia, and high B7H3 expression is positively correlated with adenocarcinoma aggressiveness, observed as overexpression in metastatic and castration-resistant disease (7–9). Further, B7H3 expression is not detected in human normal pancreas, lung, liver, kidney, colon, and heart (10). The preferential overexpression of B7H3 protein on the surface of cancer cells and the minimal expression on normal tissues makes it an ideal target for antibody-based therapeutics (11, 12), and targeting B7H3 is being widely pursued as more than 30 clinical trials are currently registered on clinicaltrials.gov.

One common strategy utilizing cell surface targets such as PSMA, B7H3, PSCA, TROP2, STEAP1, and CEACAM5 includes the development of antibody-drug conjugates (ADCs) (13, 14). ADCs combine the high target specificity of a monoclonal antibody with a cytotoxic agent for targeted killing of tumor cells. Several classes of cytotoxic drugs have been utilized in ADC designs, though most are either potent microtubule poisons or inducers of DNA damage (15). ADCs are rapidly internalized, releasing the antibody-linked payload to induce cell death. Several ADCs have been evaluated preclinically; however, only a few have been approved for clinical use due to either lack of efficacy or unacceptable toxicity, highlighting the need for strategies to define and use criteria for patient selection (16, 17). Generally, ADC-based trials have primarily focused on target antigen expression as a patient selection strategy, but absolute levels of target antigen have not been sufficient to predict response, suggesting that multiple factors be considered, including underlying mechanisms of vulnerability toward the cytotoxic payload (17). In fact, the FDA approved HER2 targeted ADC, Enhertu, has shown efficacy in the metastatic HER2-low breast cancer subtype (indication revised in August 2022) clearly suggesting a need for biomarkers other than the target antigen. Thus, a composite set of biomarkers, including target protein expression, are required to maximize clinical benefits of ADCs.

Pyrrolobenzodiazepines (PBDs) are DNA minor-groove crosslinking agents that have been used as payloads for several clinical grade ADCs (18). PBD dimers bind in a sequence-specific manner to form inter-strand crosslinks (ICLs) leading to double strand DNA breaks due to replication fork arrest (18). Determining the utility of PBD dimers for the treatment of cancers exhibiting replication stress and consequently increased potential vulnerability is a question of interest (19). Further, how tumor heterogeneity influences payload sensitivity and therapeutic efficacy has not been broadly investigated in preclinical cohorts of defined tumor types.

In this study, we evaluated a therapeutic strategy targeting B7H3 for the treatment of mPC. We profiled a spectrum of mPC tumors to assess the heterogeneity of B7H3 expression with respect to metastatic site and tumor phenotype. We tested a humanized B7H3-ADC armed with PBD payload across a range of molecularly characterized, clinically relevant mPC PDX and organoid models that reflect the diversity of human mPCs. Although we anticipated that DNA-double strand break repair defects and levels of B7H3 expression would drive B7H3-PBD-ADC responses, we observed high efficacy in (a) select B7H3-low expressing models with no apparent mutations in DNA repair pathway genes, and (b) no response in a group of B7H3^+^ adenocarcinomas. By integrating genomic and transcriptomic characteristics with B7H3-PBD-ADC response data, we uncovered additional biomarkers that represent vulnerabilities derived from more than one sensitivity or resistance pathway. These analyses demonstrate how a diverse cohort of mPCs distribute into distinct biomarker classes that reflect ADC mechanisms of action. Collectively, the results have the potential to inform patient selection for prospective trials and contribute to the interpretation of patient response and resistance outcomes.

## Results

### B7H3 is expressed across a range of mPC phenotypes and diverse metastatic sites.

**Figure 1:**
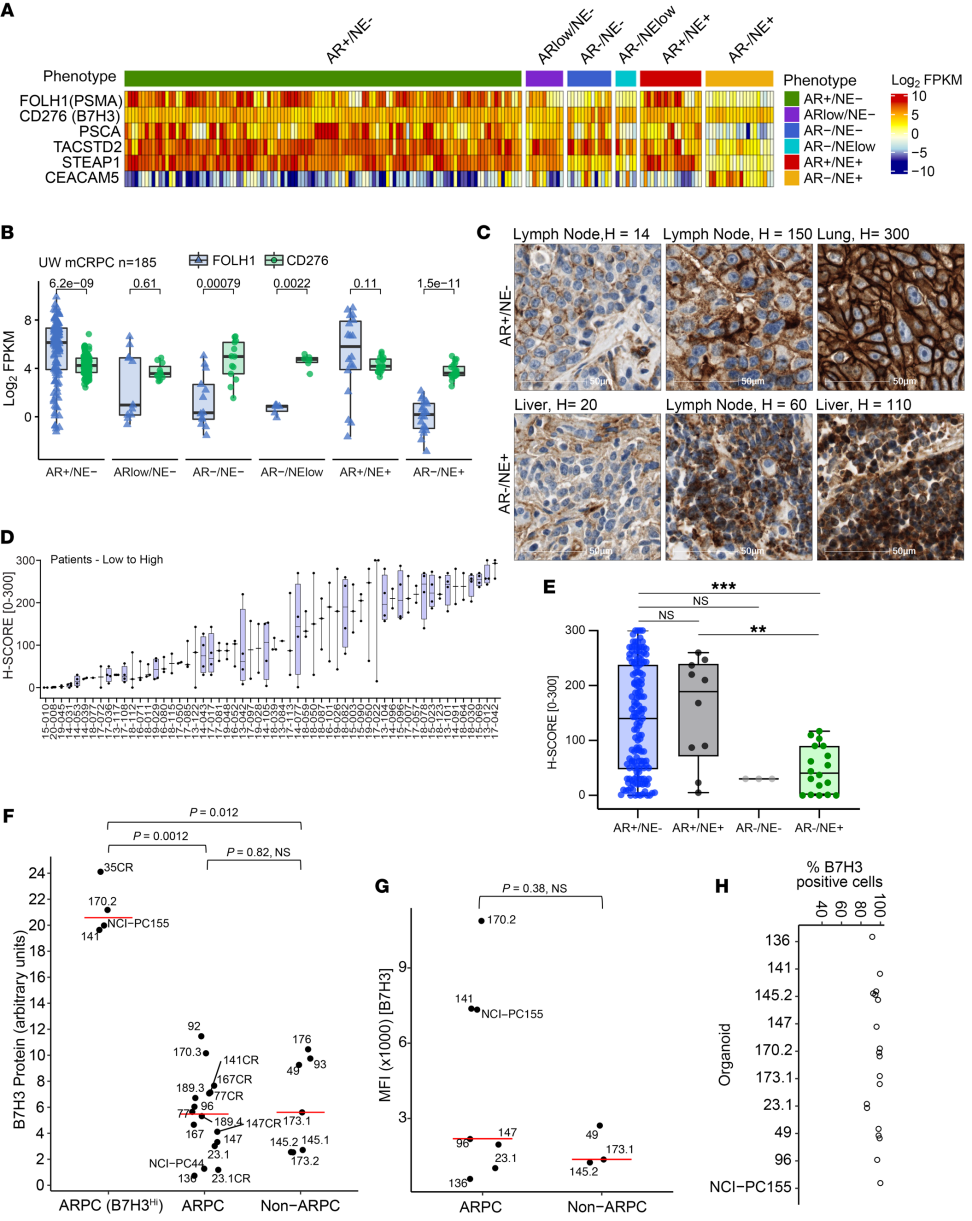
CD276/B7H3 expression in samples from patients with mPC and mPC PDX/organoid models. (**A**) *CD276/B7H3, FOLH1/PSMA, PSCA, TACSTD2/TROP2,* *STEAP1*, and *CEACAM5* transcript abundance determined by RNA-Seq analysis of 185 metastatic prostate tumors from 98 patients. Transcript levels are shown as Log_2_ FPKM. (**B**) Comparisons of *CD276/B7H3* (green dots) and *FOLH1/PSMA* (blue dots) expression by phenotypes of metastatic tumors. Groups were compared using 2-sided Wilcoxon rank tests with Benjamini-Hochberg multiple-testing correction. (**C**) IHC assessments of B7H3 protein expression. Representative staining of tumors with low, medium, and high B7H3 expression in AR^+^/NE^–^ and AR^–^/NE^+^ phenotypes. (**D**) Distribution of B7H3 protein expression in 181 metastatic tumors within and between 58 patients. (**E**) Distribution of B7H3 protein expression in mPCs categorized by phenotype (AR^+^/NE^–^; *n* = 146, AR^+^/NE^+^; *n* = 10, AR^–^/NE^–^; *n* = 3, AR^–^/NE^+^; *n* = 18, Cases not evaluated *n* = 4), **P ≤ 0.01, ***P ≤ 0.001. Wilcoxon test. (**F**) Western blot quantification of B7H3 protein expression in PDX tissue samples and 2 PDOs (NCI-PC44, NCI-PC155) by Simple Western. ARPC samples with high B7H3 expression are categorized separately in the B7H3^HI^ group. Y-axis represents CD276/B7H3 protein quantification scaled by a factor of 10. For pairwise comparison between groups, Wilcoxon test was used with *P* value adjusted using the Holm method. *P* < 0.05 was considered significant. (**G** and **H**) Flow cytometry analysis for B7H3 cell–surface expression from organoids dissociated into single cells. *P* < 0.05; significant**,** Wilcoxon test. (**G**) Median Fluorescence Intensity (MFI) and (**H**) Percentage positive cells are shown for 9 analyzed models.

To assess the potential clinical utility of targeting B7H3 as a treatment strategy for advanced prostate cancer, we evaluated the transcript abundance of B7H3 and other previously studied cell surface targets — PSMA, PSCA, TROP2, STEAP1, and CEACAM5 — in 185 tumors from 98 patients with treatment refractory mPC and across a panel of 26 mPC PDX and organoid models representing the genomic and phenotypic heterogeneity of patient tumors. The 26 preclinical models tested in this study comprise tumors of ARPC phenotype with AR signaling (intact: *n* = 13, experimentally CR: *n* = 6) as well as AR^NEG/VERY^ ^Low^ non-NEPC (*n* = 2), denoted as DNPC, (2) and SCNPC (*n* = 5), denoted at SCNPC. The latter 2 groups were collectively categorized as non-ARPC (Supplemental Figure 1A; supplemental material available online with this article; https://doi.org/10.1172/JCI162148DS1). The models included were primarily from the LuCaP PDX series (20) and also included 2 NCI mPC patient biopsy-derived organoids (PDOs) of the ARPC phenotype (21). We also categorized each patient tumor and mPC model into phenotypic categories based on gene expression signatures reflecting AR signaling and neuroendocrine (NE) pathway activity. We first quantified *CD276(B7H3)* transcript abundance in patient samples (185 tumors from 98 patients with mPC) and the above described 26 mPC models using RNA-Seq measurements. Overall, the vast majority of samples expressed *CD276* transcripts, and there was limited variation within or between mPC phenotypes compared with other targets (Figure 1, A and B, and Supplemental Figure 1B). *CD276* was also the most consistently expressed target across different mCRPC phenotypes. In contrast, other markers such as *FOLH1(PSMA)* expression varied substantially both within a phenotype and between phenotypes *(P =* 1 × 10^–9^ for the mean Log_2_ FKPM values between ARPC versus NEPC) (Figure 1B: *FOLH1* (blue), *CD276*(green)). We also evaluated the intraindividual heterogeneity of *CD276* transcript levels in multiple tumors acquired from the same patient. With few exceptions, there was a tight distribution of *CD276* expression within individuals (Supplemental Figure 1C).

We next evaluated B7H3 protein expression across a cohort of PC metastases using a tissue microarray (TMA) comprised of 181 tumors from 58 patients, with a range of 1 to 4 tumors per patient. A total of 3 tumors were not analyzed due to insufficient tumor content, leaving 178 evaluable tumors. Overall, B7H3 protein exhibited more variation compared with transcript expression: of 178 tumors evaluated, 149 expressed B7H3 (H-score > 20) and 29 lacked expression (Figure 1, C and D). B7H3 was detected across diverse metastatic sites with bone metastases exhibiting the highest levels (Supplemental Figure 1D). Tumors categorized as AR^+^/NE^–^ ARPC generally expressed higher B7H3 levels compared with other phenotypes, but a subset of AR^–^/NE^+^ SCNPC and AR^–^/NE^–^ tumors also expressed B7H3 (Figure 1E). Collectively, these results indicate that B7H3 may represent a target for antigen-directed therapeutics across a range of clinical mPC phenotypes.

Additionally, we used a quantitative immunoblot technique to determine the relative amount of total B7H3 protein expressed in mPC preclinical models. Like patient samples, we observed wide variation (more than 30-fold) in B7H3 protein levels (Figure 1F and Supplemental Figure 1E). ARPC models demonstrated a range of expression clustering as a high group (B7H3^Hi^) and an intermediate-to-low group, the latter of which overlapped in median level with the non-ARPC group (Figure 1F). There was no apparent common genotypic or phenotypic feature in B7H3^HI^ ARPC group. Consistent with patient data, B7H3 mRNA levels were not strongly correlated with B7H3 protein levels in models of either phenotype (Supplemental Figure 1F), emphasizing the minimal utility in transcriptional based assays for quantitative analyses (12). Importantly, in FACS analysis, despite variability in the median fluorescence intensity across the models tested (*n* = 9), EpCam^+^ tumor cells homogenously expressed B7H3 (80%–100% cells) at the cell surface, which makes B7H3 an ideal target for ADC based therapy (Figure 1, G and H, and Supplemental Figure 2A). B7H3 cell surface level was well correlated with total B7H3 protein (Supplemental Figure 2B).

As AR signaling is a major determinant of ARPC phenotypic subclasses, we determined the relationship of B7H3 RNA and protein to AR target gene output. However, consistent with the analyses of human mPC tumors, there was no correlation of B7H3 protein levels with AR signature scores (Supplemental Figure 2C).

### B7H3-PBD-ADC is cytotoxic for defined subclasses of prostate cancer.

**Figure 2:**
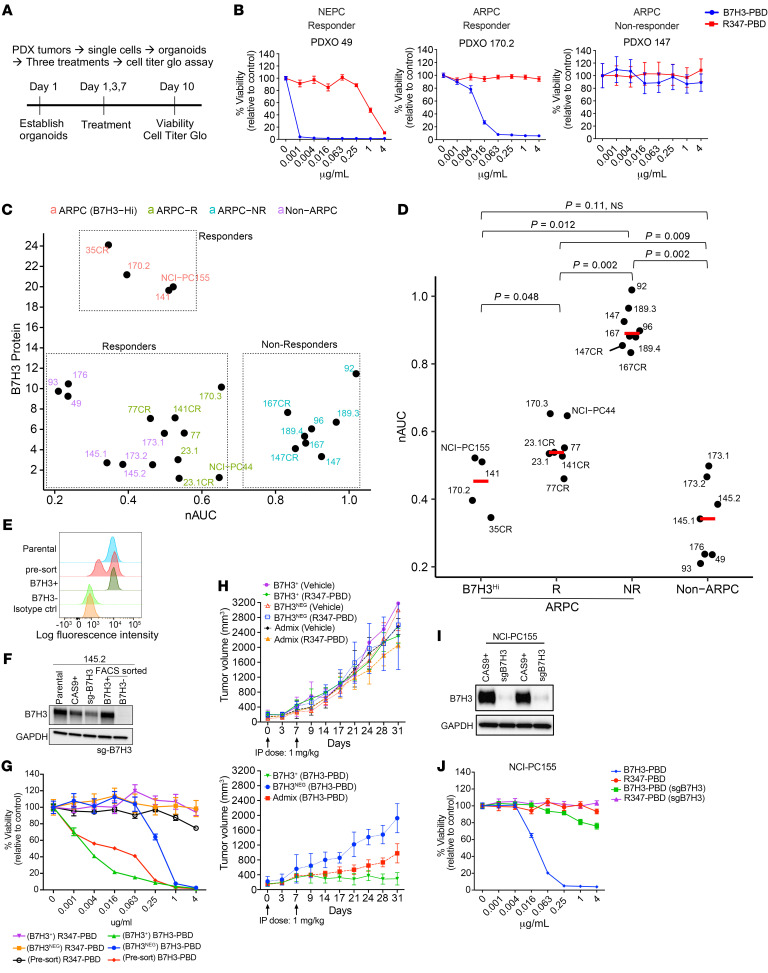
B7H3-PBD-ADC activity requires, but is not correlated with, B7H3 protein levels. (**A**) Schematic of the ex vivo drug assay. (**B**) Representative drug response curves for B7H3-PBD-ADC and R347-PBD-ADC (control ADC) in PDX-derived organoids (PDXOs) of SCNPC and ARPC phenotypes. Percentage viability was plotted relative to the control. (**C**) Comparison of B7H3-PBD-ADC response and B7H3 protein expression across the models; *n* = 26. (**D**) nAUC values for B7H3-PBD-ADC in ARPC (*n* = 19) and non-ARPC models (*n* = 7). ARPC models are categorized into 3 groups: high B7H3 expressors (B7H3^HI^) *n* = 4, responder (R) *n* = 7, and nonresponder (NR) *n* = 8. Red line indicates median nAUC for the groups. Wilcoxon test was used for pairwise comparison between groups with *P* value adjusted using the Holm method. *P* < 0.05 was considered significant. (**E**) FACS sorting strategy for selecting B7H3-KO 145.2 cells. (**F**) Western blot for FACS sorted 145.2 B7H3^+^ and B7H3^–^ (B7H3 KO) cells grown as organoids. (**G**) Dose response curves for 145.2 presorted and sorted B7H3^NEG^ and B7H3^+^ organoids treated with ADC for 10 days. (**H**) 145.2 B7H3^+^, B7H3^NEG^, and admix (mix of B7H3^+^ and B7H3^NEG^ cells in approximately equal proportion) ODXs treated with ADCs or vehicle, once weekly for 2 weeks, as indicated by arrows; *n* = 8/group, except B7H3^NEG^ (2 mice with necrotic tumors at Day 14 excluded from B7H3-PBD group), B7H3^NEG^ and admix (Vehicle group; *n* = 2 each), B7H3^+^ (Vehicle group; *n* = 4), Admix (B7H3-PBD and R347-PBD; *n* = 5 each). Average tumor volume is plotted from the day of first treatment indicated as Day 0. Top panel comparing average tumor volumes for R347-PBD and vehicle treated mice. Bottom panel comparing average tumor volumes for B7H3-PBD treated B7H3^+^, B7H3^NEG^, and admix xenografts. Wilcoxon test, *P < 0.05. (**I**) Western blot for B7H3 knockdown in NCI-PC155 organoids. (**J**) Dose response curves for NCI-PC155 organoids after B7H3 knockdown (sgB7H3 group). Error bars indicate the SEM.

We next sought to determine the efficacy of a B7H3 targeted ADC directing the genotoxic PBD (B7H3-PBD-ADC) to mPC cells. We compared the targeted delivery of PBD via B7H3-PBD-ADC, relative to the nontargeted control R347-PBD-ADC, across a panel of mPC organoids where phenotype, genotype, and B7H3 levels were established (Figure 2, A and B, Supplemental Figure 3A, and Supplemental Data File 1). All non-ARPC models were highly sensitive to the B7H3-PBD-ADC with normalized AUC (nAUC) ranging from 0.2–0.5 and IC_50_ from 0.03–2.08 ng/mL (Figure 2C and Table 1). In contrast, the ARPC models displayed a broad range of responses, with nAUC ranging from 0.3–1 and displaying less steep dose response slopes in responders compared with the non-ARPC models (Figure 2, B and C). The relative dose required for cytotoxicity in comparing targeted B7H3-PBD-ADCs and control R347-PBD-ADCs was a minimum of 1,000-fold for the most sensitive models, while the majority of models were unaffected by even the highest concentration of R347-PBD (4 mg/mL) (Figure 2B and Supplemental Figure 3, B and C).

Interestingly, there were 2 categories of ARPC responders to B7H3-PBD-ADCs, which segregated with B7H3 protein expression levels. Organoids with the highest B7H3 protein expression (LuCaPs-35CR, 170.2 and 141, and NCI-PC155) were some of the most sensitive among the ARPC models (Figure 2, C and D). There were no nonresponders among the highest B7H3 expressing ARPC models. However, many responder and nonresponder models had similar B7H3 expression, suggesting that ADC sensitivity was influenced by additional tumor biological properties. The ARPC cluster with low-to-medium B7H3 expression (B7H3^Lo^) contained responder (labeled as “R”) and nonresponder (labeled as “NR”) models. Notably, the median protein level in the ARPC (B7H3^lo^) responders was not significantly different from nonresponder ARPC models (5.61 versus 5.63, respectively, *P =* 0.58) or from responder non-ARPC models (median B7H3 levels = 5.68) (Figure 2C).

To establish specificity, we assessed whether B7H3 protein expression was necessary for ADC activity. CRISPR/Cas9 was used to generate B7H3 null organoid cells in LuCaP 145.2 and NCI-PC155 models. Because mPC organoids cannot be cloned, following B7H3 guide transduction into Cas9 expressing organoids, cells were sorted after several generations of growth based on B7H3 expression (Figure 2, E and F). The loss of B7H3 in B7H3^NEG^ LuCaP 145.2 did not result in any discernable difference in growth compared with B7H3^+^ organoids (Supplemental Figure 3D), indicating that B7H3 expression did not affect autonomous growth rate. Consistent with this, dropout screens utilizing 2 separate B7H3-directed guides in LuCaP 145.2 and LuCaP 173.1 organoids demonstrated no growth selectivity across the entire population (Supplemental Figure 3E). Importantly, loss of B7H3 in 145.2 organoids abrogated response to B7H3-PBD-ADC at concentrations less than 0.25 μg/mL in vitro, demonstrating a greater-than 400-fold increased IC_50_ compared with B7H3-WT organoids (Figure 2G). However, we observed cytotoxic effects of the B7H3-PBD-ADC at higher concentrations of 1 μg/mL and 4 μg/mL, probably resulting from B7H3^+^ contaminants in the B7H3^–^ sorted population contributing bystander effects from free PBD (11). Interestingly, organoids derived from presorted cells containing a mix of B7H3^+^ and B7H3^NEG^ cells, representative of intra-tumor heterogeneity, were almost equally sensitive as B7H3^+^ organoids, perhaps as a result of combined cell death via B7H3^+^-specific ADC activity and bystander killing effect (Figure 2G, red line). To validate B7H3-PBD-ADC specificity in vivo, we used organoid-derived xenograft models (ODXs) from sorted B7H3^+^ and B7H3^NEG^ LuCaP 145.2 organoids. To investigate the extent of bystander effect in extreme cases of heterogeneity, we also compared ADC activity in xenografts derived after mixing approximately equal proportions of B7H3^+^ and B7H3^NEG^ cells, which we labeled as admix tumors. Consistent with in vitro analysis, vehicle treated B7H3^+^ and B7H3^NEG^ ODXs displayed similar growth rates in vivo (Figure 2H, top panel). B7H3^NEG^ ODXs displayed no discernable response to the B7H3-PBD-ADC (bottom panel, blue line), whereas significant tumor regression was observed in B7H3^+^ tumors (bottom panel) compared to vehicle-treated or negative control R347-PBD-ADC–treated mice (top panel) (Figure 2H). Interestingly, admix tumors showed partial regression, although the tumor growth was significantly slower than B7H3^NEG^ ODXs after the B7H3-PBD-ADC treatment (Figure 2H, bottom panel). IHC analysis of tumors collected at the end of the study showed that B7H3^+^ cells were eradicated from both B7H3^+^ and admix tumors (Supplemental Figure 3, F and G), demonstrating specificity and suggesting that a proportion of target-negative tumor cells escape by-stander mediated killing. Finally, loss of B7H3 protein in NCI-PC155 patient-derived adenocarcinoma organoids substantially reversed B7H3-PBD-ADC sensitivity (Figure 2, I and J). These results demonstrate that the B7H3-PBD-ADC is specific for B7H3-expressing mPC organoids over a broad range of tested concentrations.

### B7H3-PBD-ADC response is associated with RB1 deficiency and replication stress.

**Figure 3:**
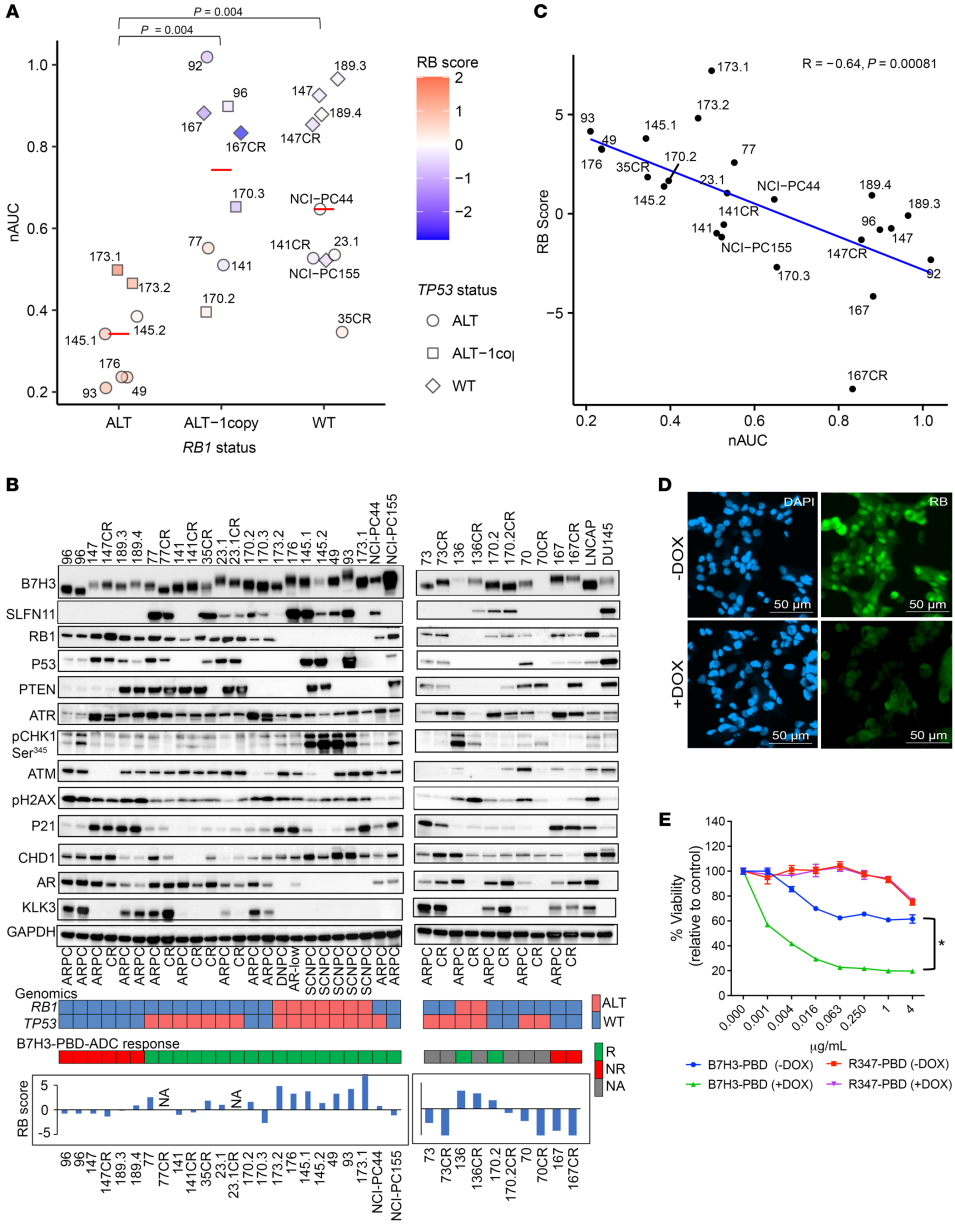
RB1 loss predicts B7H3-PBD-ADC response. (**A**) B7H3-PBD-ADC response categorized by *RB1* genomic status. Red line indicates the median nAUC for each group. *TP53* genotypes are shown as different shapes. Color indicates RB1 signature score on z-transformed scale. Wilcoxon test with *P* value adjusted using the Holm method*, P* < 0.05 was considered significant. (**B**) Immunoblot analysis of organoid models and prostate cancer cell lines for the indicated markers. Heatmap (bottom) showing *RB1* and *TP53* genomic status and B7H3-PBD-ADC response. For *RB1*, red color indicates biallelic copy loss and blue indicates WT or single copy loss. *TP53* status in red refers to alterations by biallelic inactivation or gain of function mutation and in blue indicates WT or monoallelic loss. For B7H3-PBD-ADC response; R, responsive; NR, nonresponse; NA, data not available. Bar plots for RB1 score is shown for the organoid models. (**C**) Correlation of B7H3-PBD-ADC response (nAUC) and RB1 signature score. Pearson’s correlation coefficient *r* = –0.64, *P* = 0.00081. (**D**) IF images confirming DOX-inducible knockdown of RB1 in LuCaP167 organoid model. (**E**) B7H3-PBD-ADC dose response curves in LuCaP167 (RB1^+^) organoid model expressing DOX-inducible *RB1* shRNA. Error bars indicate the SEM. *P < 0.05, Wilcoxon test.

An inspection of the model genotypes relative to B7H3-PBD-ADC efficacy to identify predictive response characteristics revealed that combined alterations of *RB1* and tumor protein 53(*TP53)* were strongly correlated with responsiveness to B7H3-PBD-ADC treatment. Indeed, *RB1* homozygous deletion and *TP53* alterations, either deletion or mutation, occur in all the non-ARPC models (Figure 3, A and B, and Table 1). Further, *RB1* function was analyzed across the models using an RB signature score that captured transcriptional networks related to RB1 functional inactivation. The RB signature score was linearly related to B7H3-PBD-ADC sensitivity, as measured by AUC in organoid models, suggesting not only a categorical relationship but also a quantitatively determined sensitivity (Figure 3C).

We experimentally validated *RB1* levels as a determinant for B7H3-PBD-ADC responses by depletion via doxycycline-induced shRNA in the nonresponsive, *TP53*^WT^*RB1*^WT^ LuCaP 167 ARPC organoid model (Figure 3D and Supplemental Figure 4A). Consistent with the results across the various models, *RB1* depletion resulted in increased B7H3-PBD-ADC cytotoxicity in LuCaP 167 organoids, demonstrating *RB1* levels as an independent factor in determining B7H3-PBD-ADC sensitivity (Figure 3E).

Since models with *RB1* loss showed exceptional response to the B7H3-PBD-ADC, despite having low levels of B7H3 protein, we also tested the influence of this underlying genomic vulnerability on the in vitro activity of free PBD dimer. Indeed, increased sensitivity to free PBD dimer was observed in *RB1*-loss models compared with *RB1*^WT^ models, suggesting that the genotoxic effect of PBD induced inter-strand DNA crosslinks is potentiated by loss of functional *RB1* (Supplemental Figure 4B). Thus, the response to the B7H3-PBD-ADC is substantially governed by the interplay between tumor characteristics and the mechanism of action for the attached payload and not necessarily by the density of the targeted antigen.

**Figure 4:**
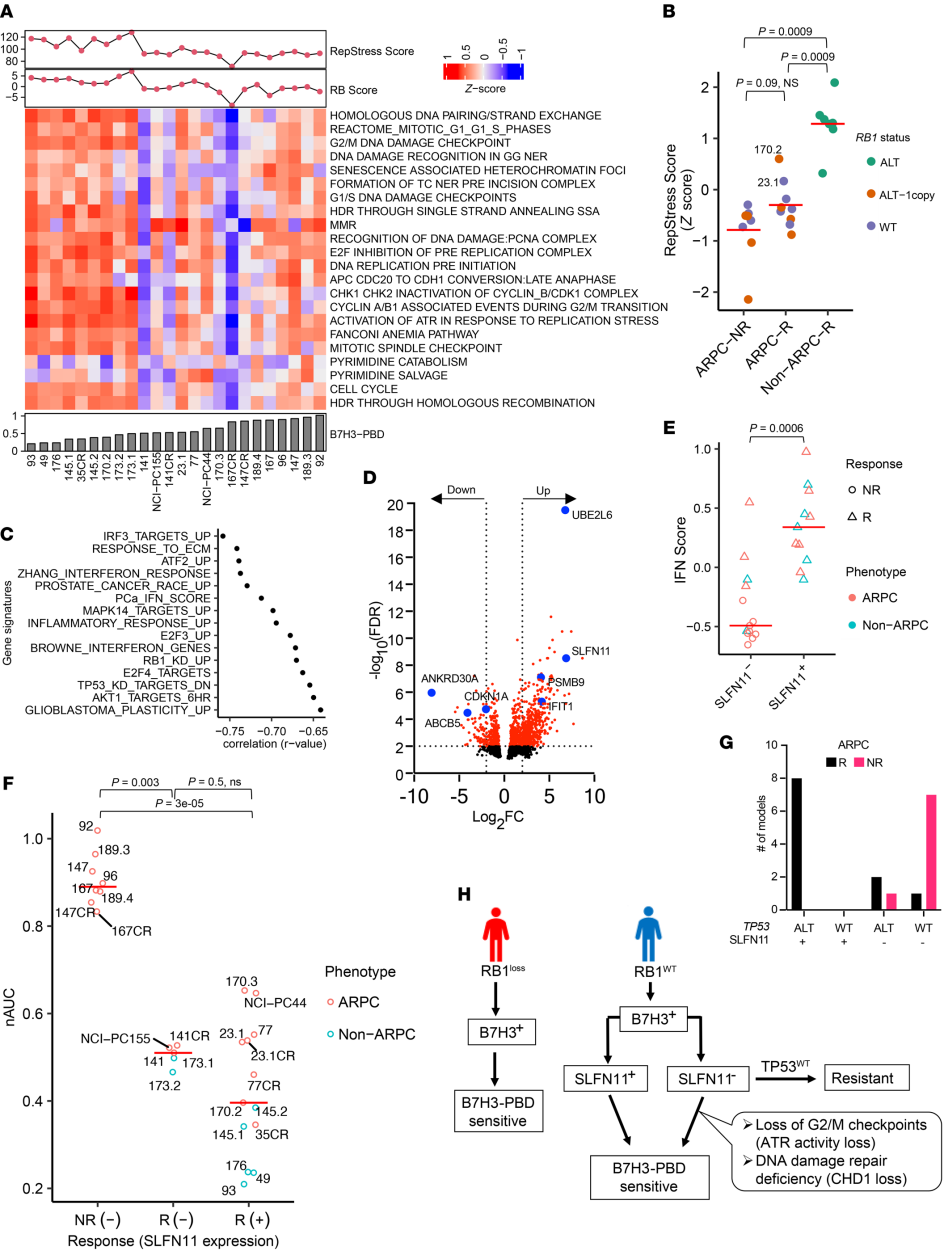
Contributing biomarker subclasses of B7H3-PBD-ADC sensitivity. (**A**) Heatmap of the pathways contributing to the RepStress signature score. Organoid models are ranked from left to right based on increasing B7H3-PBD nAUC (bottom panel). Top panel showing RepStress and RB signature scores. (**B**) Comparison of z-transformed RepStress score in AD nonresponders (ARPC-NR), ARPC responders (ARPC-R), and non-ARPC responders (NonARPC-R). Color indicates *RB1* genotype. (**C**) Univariate correlation analyses between nAUC and MsigDB gene signatures, including refined IFN signature score for prostate cancer labeled as PCa_IFN_Score. Spearman correlation coefficient is shown for top significant gene sets. FDR ≤ 0.05, Benjamini and Hochberg method for multiple hypothesis test correction. (**D**) Volcano plot for differentially expressed genes between B7H3-PBD-ADC–responsive ARPC models versus nonresponsive ARPC models. Dotted lines are shown for log_2_ fold change of –2 and 2 at FDR ≤ 0.01. (**E**) Comparison of IFN score with SLFN11 expression. (**F**) B7H3-PBD-ADC response categorized by SLFN11 expression. Red line indicates median nAUC for each group. NR, nonresponder; R, responder; +/- indicates SLFN11 expression. *P*<0.05; significant, Wilcoxon test. (**G**) Distribution of ARPC models based on *TP53* genomic status and SLFN11 expression. (**H**) Schematic of proposed biomarker based therapeutic decisions for B7H3-PBD-ADC treatment of patients with mPC. For multiple group comparisons in panel **B** and **F**, *P* values were determined by Wilcoxon test and adjusted using the Holm method.

*RB1* loss is associated with replication stress, and, not unexpectedly, replication-stress induced pathways also correlated with the strength of B7H3-PBD-ADC responses. We used a replication stress signature score (RepStress score), modified for prostate cancer based on DNA damage and cell cycle pathways involved in replication stress, and analyzed association with B7H3-PBD sensitivity (Figure 4A). Across all models, including ARPC and non-ARPC groups, a high RepStress score was significantly correlated with higher sensitivity to B7H3-PBD-ADC (Supplemental Figure 4C), a finding that is strongly determined by loss of RB functionality (Supplemental Figure 4D). Moreover, B7H3-PBD-ADC showed greater efficacy in vitro, compared with other known RepStress-sensitive drugs, including Topotecan, Cisplatin, and Mitomycin C (Supplemental Figure 4, E and F). For the ARPC models only, the majority of the ARPC responders (“ARPC-R”) had similar replication stress scores as non-responders (“ARPC-NR”), with the exception of 2 responder RB1^WT^ models (LuCaPs 23.1 and 170.2) that demonstrated RepStress scores above the average (Figure 4B). This implies that PBD sensitivity of mPC with functional RB1, usually ARPC, is determined by factors in addition to replication stress.

### Schlafen family member 11 expression and TP53 status are predictors of B7H3-PBD-ADC response in RB1 functional prostate cancer.

To address broadly predictive biomarkers in ARPC with functional *RB1*, the most common clinical phenotype of mPC, we analyzed differentially expressed signaling pathways in ARPC-R versus ARPC-NR. Correlation analysis with single sample gene set enrichment scores identified interferon response gene signatures as the topmost correlated signaling pathways for B7H3-PBD-ADC sensitivity (nAUC) for ARPC models (Figure 4C). To further delineate the role of specific interferon stimulated genes (ISGs) that may be contributing to B7H3-PBD-ADC response, we performed differential expression analyses comparing ARPC responders (*n* = 9) and nonresponders (*n* = 8), which revealed several ISGs among the top 20 differentially upregulated genes in responders, including *UBE2L6*, *PSMB9*, and schlafen family member 11 (*SLFN11),* while *CDKN1A* and *ABCB5* were notably downregulated (Figure 4D and Supplemental Data File 2). Upregulation of SLFN11 in ARPC-R models was of particular interest, as it is a known sensitizer for toxicity mediated by specific DNA damaging agents (22, 23). *SLFN11* is a nonclassical IFN-response gene, and indirect effects of IFN signaling likely contribute to contextual SLFN11 expression (22). As expected, in the phenotypically heterogeneous models analyzed here, SLFN11^NEG^ models demonstrated a significantly lower median IFN signature score than SLFN11^+^ models (Figure 4E). We categorized B7H3-PBD-ADC response based on SLFN11 positivity or negativity. Consistent with differential expression analysis, SLFN11 expression predicted response in 8 of 8 ARPC models, demonstrating SLFN11 as a robust positive biomarker in the models analyzed here (Figure 4F). Of note, we observed that enrichment for WT TP53 alleles was a common molecular characteristic among 7 of 8 SLFN11^NEG^ nonresponders, suggesting that SLFN11 expression may be linked to TP53 mutation status (Figure 4G and Table 1). It should be noted that a small number of SLFN11^NEG^ models were also responsive to the ADC (3 responders out of 11 SLFN11^NEG^ ARPC models), suggesting that lack of SLFN11 expression is not always predictive and that other nonoverlapping mechanisms/biomarker classes also lead to sensitivity (Figure 4F) in ARPC, as described below. Similarly, the majority (6 of 9) of RB1-deficient models were SLFN11^+^ (Figure 3B), but RB1 deficient models were responsive to B7H3-PBD-ADC independent of SLFN11 expression, demonstrating that replication stress predicts sensitivity even in the absence of SLFN11 (Figure 4F, in the R(–) group; 173.1 and 173.2).

In B7H3 expressors*,* *RB1* loss and/or replication stress and/or SLFN11 expression predicted the responses of most tumors to B7H3-PBD-ADC treatment, but there were outliers where these features did not discriminate outcomes. For example, LuCaPs 141 and 141CR, and NCI-PC-155 were categorized as *RB1*^WT^ and SLFN11^NEG^ responders (Figure 3B and Figure 4G). We analyzed known genomic vulnerabilities associated with inability to repair ICLs and expression of previously known transporters of PBD. None of the previously identified transporters of PBD (*ABCG2, ABCB1, ABCC2,* and *SLC46A3*) correlated with responsiveness in the extensive mPC cohort tested here (Supplemental Figure 4G). Because ATR loss of function is a sensitizing factor for PBD responsiveness (24), we performed an ATR activation assay in response to DNA damage in ARPC organoids of B7H3-PBD-ADC responders: LuCaP 77 (*RB1*^WT^, SLFN11^+^), LuCap 141CR and NCI-PC155 (*RB1*^WT^, SLFN11^NEG^), and the nonresponder LuCaP 167 (*RB1*^WT^, SLFN11^NEG^). Functional ATR was evident in response to topotecan and B7H3-PBD-ADC in the responder LuCaP 77 and LuCaP141CR models. However, ATR was reduced to near undetectable levels in responder NCI-PC155 and nonresponder LuCaP 167 (Supplemental Figure 5, A–D). Consistent with nonresponsiveness, LuCaP 167 showed no evidence of PBD-mediated DNA damage. By contrast, NCI-PC155 had clearly detectable topotecan and B7H3-PBD-ADC induced DNA damage (γH2AX) compared with LuCaP 167 (Supplemental Figure 5, A and B). These data for NCI-PC155 are consistent with a mechanism whereby very weak ATR signaling enhances DNA damage-induced death.

As the SLFN11^NEG^ LuCaP 141CR model had an intact ATR pathway, we considered other DNA repair biomarkers. Importantly, we observed loss of CHD1 protein in LuCaPs 141 and 141CR (Figure 3B). CHD1-deficient cells are generally hypersensitive to DNA cross-linking agents because of defects in homologous recombination–mediated repair, and, in clinical mPC, *CHD1* mutations are statistically associated with a predicted homologous recombination deficiency (HRD) (25, 26). These data are consistent with a responsive phenotype due to a DNA repair deficiency for LuCaPs 141 and 141CR, despite an *RB1^WT^* genotype and lack of SLFN11 expression (Figure 4H).

Indeed, our analysis of ATR activity and CHD1 loss is limited by the number of available models, which are derived from CRPC patient populations in which *ATR* and *CHD1* mutations occur at a frequency of under 5%. Although the proposed mechanisms for responsiveness in these *RB1^WT^*SLFN11^NEG^ models require validation, the observation of their existence is notable and directs future studies to investigate relatively infrequent DNA repair deficiency-mediated responsive mechanisms.

### In vivo models of mPC validate organoid response classes to B7H3-PBD-ADC therapy.

**Figure 5:**
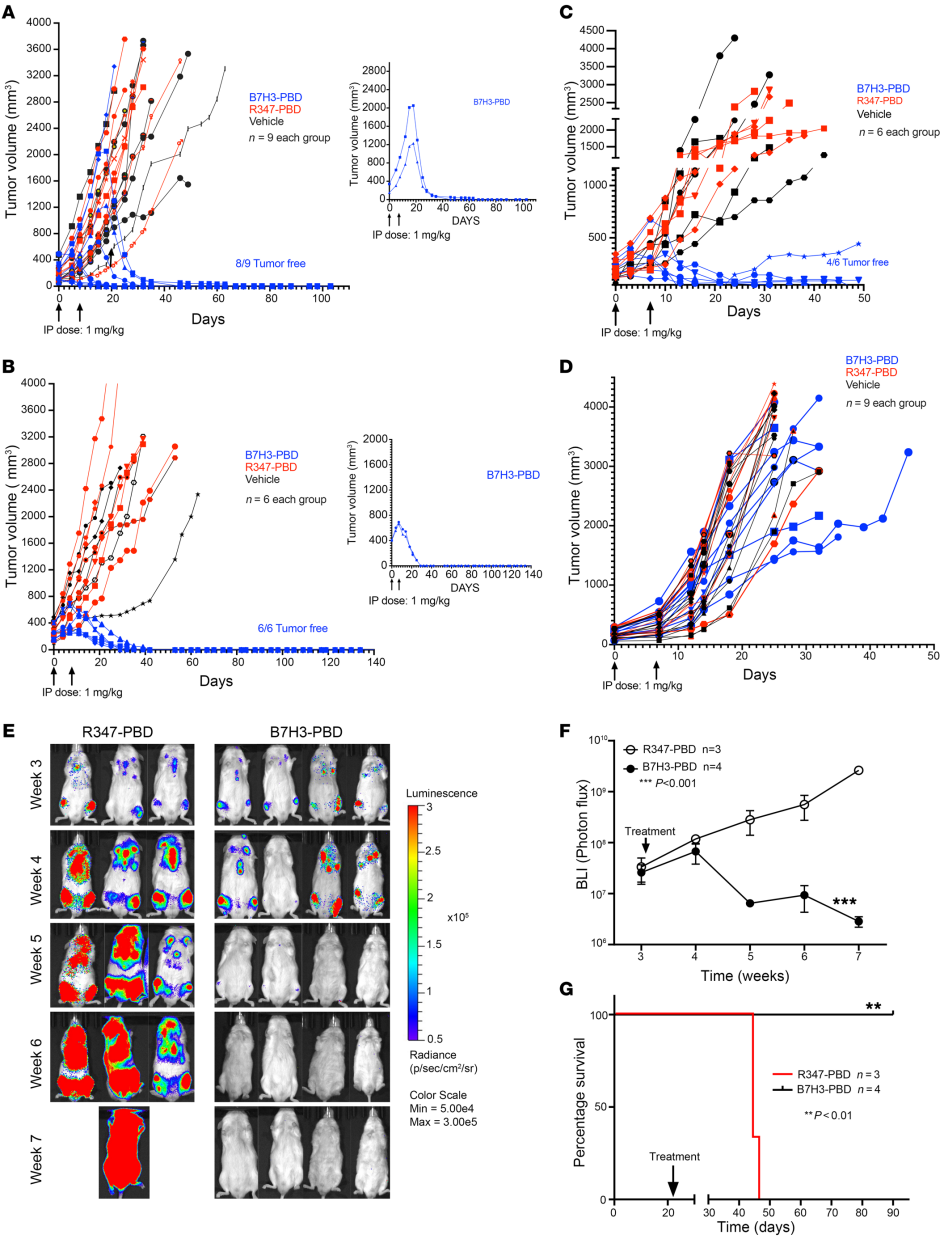
Prostate cancer organoid-derived biomarkers predict in vivo tumor responses in preclinical trials of the B7H3-PBD-ADC. (**A**–**D**) Tumor response to B7H3-PBD-ADC (1 mg/kg), R347-PBD-ADC (1 mg/kg) or vehicle in the 4 selected LuCaP models based on identified biomarkers (**A**) LuCaP 145.2 (SCNPC phenotype; RB1^loss^, SLFN11^+^, IFN score^Hi^), *n* = 9 / group. (**B**) LuCaP 136 (ARPC phenotype; RB1^loss^, SLFN11^–^, IFN score^lo^), *n* = 6/ group. (**C**) LuCaP 77 (ARPC, RB1^+^, SLFN11^+^, IFN score^medium^) *n* = 6 /group. (**D**) LuCaP 167 (ARPC, RB1^+^, SLFN11^–^, IFN score^lo^) *n* = 9 /group. Right panels for **A** and **B** display antitumor activity of B7H3-PBD-ADC in mice with large established tumors (145.2; > 1,000 mm^3^ and 136; > 650 mm^3^). Tumor volume measurements (mm^3^) are shown from the time of first treatment. Arrows indicate once weekly dose for 2 weeks. (**E**) BLI of LuCaP 136 metastases following treatment with B7H3-PBD-ADC (*n* = 4) or R347-PBD-ADC (*n* = 3). Mice were treated once weekly for 2 weeks. (**F**) Average BLI for treated mice from the time of first treatment. Means ± SD, 2-way ANOVA test; *P* < 0.001. (**G**) Kaplan-Meier survival analysis for the B7H3-PBD-ADC and R347-PBD-ADC treated LuCaP 136 metastases. Log-rank test (*P* < 0.01).

Based on the in vitro B7H3-PBD-ADC response data and analysis of potential biomarkers, we evaluated the efficacy of B7H3-PBD-ADC treatment in preclinical trials of PDX models representative of different phenotype and genotype mPC categories defined by organoid studies: *RB1*^NULL^ (SLFN11^+^ or SLFN11^–^) and *RB1*^WT^ (SLFN11^+^ or SLFN11^–^) tumors (Figure 5). We randomized mice implanted with PDX lines to treatment with 2 i.p. doses of 1 mg/kg B7H3-PBD-ADC or control R347-PBD-ADC, given weekly for 2 weeks. The SCNPC LuCaP 145.2 (*TP53*^ALT^*RB1*^–/–^SLFN11^+^) line showed a complete and durable response to B7H3-PBD-ADC treatment (Figure 5A). Control R347-PBD-ADC and vehicle-treated mice had similar tumor growth indicating no apparent nonspecific effects of PBD at a 1 mg/kg dose. Remarkably, no tumors were detected in 8 out of 9 B7H3-PBD-ADC treated mice 3 months after therapy. Furthermore, 2 of the large established tumors (over 1,000 mm^3^) were completely regressed with just 2 doses of B7H3-PBD-ADC (Figure 5A, right panel).

Similarly, the LuCaP 136 ARPC PDX (*TP53*^–/–^*RB1*^–/–^SLFN11^–^) showed complete durable response in all 6 mice treated with B7H3-PBD-ADC compared with control R347-PBD-ADC–treated or vehicle-treated mice (Figure 5B). LuCaP 136 was not evaluable for in vitro responses due to poor growth characteristics beyond 1 week, but the *RB1* loss phenotype predicted in vivo responsiveness. Again, B7H3-PBD-ADC treatment resulted in a remarkable decrease in large tumor burden (approximately 800 mm^3^) in 2 mice (Figure 5B, right panel). 100% of the mice were tumor free for more than 4 months after treatment. Further, B7H3-PBD-ADC treatment of the ARPC LuCaP 77 xenograft (*RB1*^WT^SLFN11^+^) also showed a durable response relative to R347-PBD-ADC treated mice (Figure 5C). In contrast, the ARPC LuCaP 167 xenograft (*RB1*^WT^SLFN11^–^), which did not respond to B7H3-PBD-ADC in vitro, also showed no significant tumor regression with B7H3-PBD-ADC treatment compared with R347-PBD-ADC–treated or vehicle-treated mice (Figure 5D). Thus, the treatment responses assessed via organoid assays accurately predicted the matching in vivo responses to B7H3-PBD-ADC.

Because the majority of clinical mPCs progress following ADT and ARSI treatment with the retention of AR signaling, and patients often harbor bone metastases, we further tested B7H3-PBD-ADC activity against a late-stage ARPC bone metastasis model. We developed a model system whereby intracardiac injection of luciferase-tagged AR^+^ LuCaP 136 (*TP53*^–/–^*RB1*^–/–^SLFN11^–^) tumor cells colonized bone with 100% efficiency. The majority of metastases were located in the vertebrae. Other sites included calvaria, sternum, and long bones, as well as less than 10% of tumor burden in soft tissue (adrenals and liver). Using bioluminescence imaging (BLI) to monitor the growth of tumor metastasis, mice were grouped into 2 arms of equal average metastasis burden. B7H3-PBD-ADC treatment once weekly, for 2 weeks, substantially reduced tumor burden resulting in no detectable metastasis compared with control R347-PBD-ADC–treated mice (Figure 5, E and F). Overall, B7H3-PBD-ADC treatment resulted in long-term metastasis-free survival in 100% of the treated mice (Figure 5G). These data extend B7H3-PBD-ADC efficacy to tumors residing in clinically relevant microenvironments.

We evaluated the safety profile of B7H3-PBD-ADC both in vitro and in vivo. Body weights were unaffected by the ADC in all in vivo preclinical trials (Supplemental Figure 6, A–D). In addition, we performed full necropsy on day 8 and day 30 after treatment to evaluate acute and delayed in vivo toxicity, respectively, in the LuCaP 136 PDX model after administrating the ADC (*n* = 3 per group). Histopathology analysis demonstrated a reasonable safety profile with no obvious changes in gross pathology. H&E-stained sections of liver, heart, lung, brain, adrenal gland, kidney, small intestine, spleen, and prostate collected on day 8 or day 30 after treatment from all animals were examined. Treatment-related microscopic changes were limited to minimal small intestine crypt apoptosis in R347 or B7H3-treated animals on day 8 after treatment (Supplemental Figure 6E). This change had recovered by day 30 after treatment. All remaining microscopic changes were similar between untreated and treated animals or consistent with common background findings in mice. Further, human IgG immunoreactivity was not observed in any study tissues examined from vehicle, R347-PBD-ADC, or B7H3-PBD-ADC–treated animals collected at day 8 or day 30 after treatment, suggesting no accumulation of ADC in normal mouse tissues. Additionally, we tested the B7H3-PBD-ADC and free PBD payload in organoids derived from normal mouse prostate and normal human liver cells (21). Consistent with the in vivo results, the ADC showed no toxicity in human liver cells (Supplemental Figure 6F) or normal mouse prostate organoids (Supplemental Figure 6G), whereas free PBD affected viability of normal organoids only at substantially higher concentrations (IC_50_ = ~2–5 nM) than that observed for responder tumor models (IC_50_ < 0.03 pM) (Supplemental Figure 4B and Supplemental Figure 6G).

### Distribution of biomarkers in clinical samples.

We related the results presented here (Figure 4H) to the distribution of biomarkers in clinical CRPC by analyzing the SU2C mPC data set, considering samples with over 30% tumor content (303 samples were included) (Supplemental Figure 7). Within these samples, approximately 10% demonstrated homozygous *RB1* alterations (3, 27). The influence of SLFN11 upon drug sensitivity has been associated qualitatively with presence of the RNA and protein (19, 23, 24). From the SU2C RNA-Seq data, we estimated that about 40% of *RB1* intact samples expressed SLFN11 (see methods) (Supplemental Figure 7). Further, of the remaining *RB1*^WT^/*SLFN11*^NEG^ samples (*n* = 153), about 4% of patients (6 of 153), demonstrated CHD1 or ATR loss, whereas 91 out of 153 had *TP53*^WT^ and no ATR/CHD1 alterations. In summary, considering any single biomarker as predictive for response, approximately 50% of the SU2C cohort could have been considered further for B7H3-PBD-ADC treatment, pending the determination of B7H3 protein expression.

## Discussion

Here, we present importance of biomarker stratification within tumors from patients with mPC using an organoid/PDX platform exemplifying the extensive genomic and phenotypic heterogeneity of clinical disease. We evaluated the rapidly developing therapeutic modality of antibody-directed cytotoxics, specifically B7H3-targeted PBD, a DNA interstrand cross-linking agent. Novel approaches to treatment of advanced mCRPC are needed as acquired resistance to ARSIs occurs with near universal frequency. We determined that B7H3 is expressed across a diverse spectrum of late-stage treatment-resistant mPC genotypes and phenotypes as well as across metastatic sites, which extends prior analyses of B7H3 in prostate cancer (7–9, 28).

B7H3 expression is necessary for targeted delivery and ADC response, as demonstrated by the loss of responsiveness upon CRISPR/CAS9–mediated B7H3 loss (Figure 2, E–J). Importantly, however, B7H3 expression alone is not predictive, as several B7H3^+^ adenocarcinomas were resistant to treatment. Here, and with other ADCs, response biomarkers are required to stratify patients to optimize efficacy and minimize ineffective drug exposure. PBD delivered via B7H3-ADC directed exposure exhibited a range of in vitro and in vivo activity against mPC organoid models that reflect underlying biology described by genotypic and phenotypic markers. Molecular interrogation revealed biomarker-defined classes of responsive models including: (a) *RB1/TP53* loss of function and associated replication stress observed predominantly in SCNPC and in some highly aggressive adenocarcinomas, (b) SLFN11 expression observed in a subset of *RB1* WT/*TP53* altered adenocarcinomas, and (c) specific DNA repair mutations (including *ATR* and *CHD1)* that impact PBD-initiated DNA damage. Thus, multiple vulnerabilities distinct from AR-dependent survival and, including but expanded beyond, homologous recombination deficiencies targeted with PARP inhibitors can be exploited using PBD-based therapeutics. The mPC PDX/organoid cohort analyzed here approximately replicates the distribution of identified baseline biomarkers, *RB1* and *TP53,* mutations as well as SLFN11 expression, observed in the large SU2C CRPC clinical data set. We anticipate that the classification and associated rationale for biomarker-identified organoids here largely reflects the potential response spectrum in clinical mPC and will inform the design and impact the overall efficacy of prospective trials much more accurately than the prior therapeutic analyses with limited numbers of in vitro growth–selected mPC cell line models previously available.

A category of biomarkers associated with B7H3-PBD-ADC responses involve a vulnerability to replication stress. A unique conclusion of this study is that mPCs with functional alterations in *RB1/TP53* are highly sensitive to B7H3-PBD-ADC irrespective of their histological phenotype. Defects in *RB1* and *TP53* pathways are known to promote enhanced replication stress (27), and additional blockage of replication forks by PBD-induced ICLs likely contributes to its potent cytotoxicity. It will be useful to determine whether ADCs with PBD payloads have the potential to be efficacious in other cancer types with high replication stress phenotypes. Although clinical studies have demonstrated that *TP53/RB1*–deficient SCNPCs, as well as small cell lung cancer, generally show high response rate to platinum chemotherapy, many patients respond for relatively short periods of time, and patients almost universally succumb to recurrent disease (29, 30). Here, *TP53/RB1* deficient models of various histological types demonstrated highly efficacious complete responses to B7H3-PBD-ADC. This suggests that B7H3-PBD-ADC may be considered for treatment of *RB1*-deficient cancers following platinum resistance.

SLFN11 expression was positively predictive of sensitivity to B7H3-PBD-ADCs. SLFN11 is a DNA/RNA helicase that is actively recruited to sites of DNA damage and appears to irreversibly block stressed replication forks, leading to the hypothesis that SLFN11 is a dominant inhibitor of stressed replication fork repair. SLFN11 is likewise a useful biomarker for response to a variety of other therapeutics that target enhanced replication stress, including topoisomerase and PARP inhibitors as well as platinum chemotherapeutics (19, 23). Of note for the present study of mPCs, we found that a cell-autonomous IFN score was positively associated with expression of SLFN11, a noncanonical IFN-response gene (22), and negatively correlated with the presence of a WT *TP53* allele. Future studies to establish the mechanistic basis of this observation could hold importance in utilizing multiple associated biomarkers to optimize therapeutic predictions and to gain insight into the physiological context of SLFN11 activity.

The direct or indirect repair of PBD-initiated DNA interstrand crosslinks, a major consequence of which is the deleterious blockage of replication forks, is an anticipated class of mechanistic biomarkers. Although further confirmatory studies are required, we present preliminary data that loss of CHD1 appears to sensitize toward B7H3-PBD-ADCs killing, as observed for the SLFN11^NEG^/*RB1*^WT^ adenocarcinoma model, LuCaP 141. By contrast, the LuCaP 96 model, which displays loss of function for *BRCA2,* a classical HRD protein, was nonresponsive to B7H3-PBD-ADCs. It is likely noteworthy that LuCaP 96 expressed WT *TP53*, and previous analyses of *BRCA1/2–*deficient breast cancer PDXs have suggested that approximately 25% are nonresponsive to PBD, a phenotype that may be associated with a less compromised HRD resulting from minimal mutations to additional DNA repair proteins, including *TP53* (31, 32). In addition, we characterized a naturally occurring, PBD-responsive, SLFN11^NEG^ *TP53*^WT^ ATR loss-of-function model, NCI-PC155. This observation is from a natural patient-derived model, providing further support for the conclusions drawn from experimentally selected SLFN11^NEG^ tumor models where inhibition of ATR was synthetically lethal (19, 24).

In summary, B7H3-PBD-ADCs deliver a potent ICL agent with substantial antitumor effects toward subtypes of mPC, refractory to standard-of-care treatment regimens. Several acquired resistance mechanisms in mPC appear sensitive to the action of PBD, supporting further evaluation as a therapeutic strategy for cancers that are refractory to currently approved agents. Collectively, our analyses suggest that mPCs vulnerable to PBD action can be identified by a composite set of biomarkers that address multiple histological phenotypes, including adenocarcinomas and SCNPC, and utilize genomic markers (*RB1, TP53, CHD1,* and *ATR)* and/or phenotypic markers (B7H3, SLFN11, and RepStress scores). The approach reported here for identifying biomarkers of vulnerability to a particular cancer therapeutic using an extensive cohort of diverse models of mPC has the potential to increase the reliability of translation to the clinic as well as to provide insights into mechanisms that underlie the more precise allocation of therapy.

## Methods

### Study design.

The objective of this study was to evaluate B7H3 expression and treatment efficacy of the B7H3 ADC armed with a PBD warhead (SG3315) in a diverse spectrum of PDX/organoid preclinical models of mPC. An additional objective was to identify predictive biomarkers of B7H3-PBD response. In total, 37 mPC organoid models were evaluated for B7H3 expression. At least 26 of 37 organoid models that can be grown in vitro for a minimum of 10 days were tested with the ADCs (Supplemental Figure 3A). Gene expression data for 24 out of 26 organoid models (not available for 23.1CR and 77CR) was utilized to identify correlates of PBD response.

For in vivo studies, the sample size for each experiment is indicated in the figure legend. The animal caretaker who assessed and treated animals and measured tumors was blinded to the intervention. All other investigators were not blinded for any experiments.

### Properties of anti-B7H3 ADC.

B7H3-PBD-ADC is composed of a Human IgG1 anti-B7H3 antibody, site-specifically conjugated via a cathepsin-cleavable valine-alanine (val-ala) linker to a PBD dimer warhead SG33115 (patent WO 2015/052322). The average number of drugs linked to each antibody molecule (DAR) was 2. Anti-B7H3 antibody (see supplemental methods for details) is non-mouse cross reactive (also shown in Supplemental Figure 6F) and binds to huB7H3 but not huB7H4 (with approximately 29% homology among the closest family homologs).

### RNA-Seq Analysis of mPC tumors.

RNA isolation and sequencing of 185 UW mPC tumors from 98 patients were performed as described previously (2). Sequencing reads were mapped to the hg38 human genome using STAR.v2.7.3a. All subsequent analyses were performed in R. Gene level abundance was quantitated using GenomicAlignments and transformed to log_2_ FPKM. Groups were compared using 2-sided Wilcoxon rank tests with Benjamini-Hochberg multiple-testing correction. To evaluate the expression of B7H3 from tumors within the same patient, boxplots of transcript levels from tumors from the same patient were filtered to include only patients with at least 2 tumors profiled (149 tumors from 62 patients) and are ordered by perpatient median log_2_ FPKM gene expression.

### IHC.

TMA slides consisting of triplicate cores from 181 metastatic prostate sites representing 58 donors were provided by the genitourinary lab at the University of Washington. Automated IHC was performed on the VENTANA Discovery Ultra (Ventana Medical Systems Inc.) autostainer. Details of the method for IHC staining are provided in the supplemental methods.

The TMA slides were scanned at a ×40 magnification using the Ventana DP 200 instrument (VMSI) and visualized with HALO (Indica Labs). Staining intensity was evaluated by a pathologist. The H-score was determined by multiplying the percentage of positive cells at each intensity level (0 denotes no staining, 1 denotes weak staining, 2 denotes moderate staining and 3 is strong staining) by its respective intensity level. The weighted scores were totaled, resulting in a composite score ranging from 0–300. The triplicate scores for each site were averaged to generate an averaged H-score for each site.

### Organoid culture.

LuCaP PDX tissue samples were processed into single cells, depleted for mouse cells using mouse cell depletion kit (Miltenyi Biotec; manufacturer’s protocol), and plated as organoids according to our previously described methods (21). Organoids were grown in 5% Matrigel on ultra-low attachment plates/dishes (Corning) for short-term drug treatment and flow cytometry analysis. Previously defined organoid culture media (21) was supplemented with 5 ng/mL Neuregulin 1 (Peprotech3) to potentiate the growth of adenocarcinoma organoid models.

### Pharmacological agents.

Two batches of ADCs (B7H3-PBD or R347-PBD) were received from AstraZeneca. Organoid responses with an individual batch of the ADC are shown in Supplemental Figure 3A. No major difference in drug response was observed. The R347 is a control human IgG1 antibody that does not bind human proteins and serves as the isotype control antibody-drug conjugate. Free PBD payload was purchased from MedChemExpress. Topotecan (S9321), ATR inhibitor, Berzosertib (S7102), Carboplatin (S1215), Cisplatin (S1166), Mitomycin C (S8146), and Doxorubicin (S1208) were purchased from Selleckchem.

### Organoid drug response assays.

PDX tumors and organoids were dissociated into single cells, mixed with Matrigel, and plated at 1,000 cells/well in 384-well poly d-lysine coated plates (Corning). Drugs were serially diluted in organoid culture media and a total volume of 30 μL per well was added. Organoids were treated with the small molecule inhibitors, B7H3-PBD or R347-PBD (control ADC) 3 times for 10 days (Figure 3A). Thereafter, cell viability was quantified with CellTiter Glo 3D. Luminescence was measured using Infinite M200 Pro microplate reader (Tecan). Each experiment included 5 replicates. All treatments were repeated in 2 or more biologically independent experiments. nAUC was calculated for each experiment as a ratio of AUC for B7H3-PBD and R347-PBD dose-response curves. Median nAUC and interquartile range was then calculated across independent experiments for each organoid model. Median nAUC was used for all further analysis. GraphPad Prism 9 was used to calculate IC_50_ and maximum drug effect (MaxR) which is shown as percentage viable cells at the highest concentration of 4 μg/mL.

### Simple Western protein quantification.

LuCaP PDXs’ tissue lysates were prepared using RIPA buffer containing protease and phosphatase inhibitors. Protein samples were separated based on size using a capillary-based automated protein analysis system (Protein Simple Western Technology, Peggy Sue). Protein separation, immunodetection, and analysis steps were performed automatically by the protein analysis system. Protein bands were analyzed with the Compass software. The observed band size of B7H3 protein was variable between 120–150 kDa. GAPDH was used as the internal control. The B7H3 protein was quantified by normalizing B7H3 band intensity to the housekeeping protein, GAPDH. For the figures, the values were scaled by a factor of 10. See supplemental methods for additional details.

### Immunoblots.

LuCaP PDX tissue lysates were prepared as above. Organoid pellets were lysed in warm 1% SDS buffer with protease and phosphatase inhibitors. To shear the released DNA/RNA and reduce sample viscosity, 25 U/μl Benzonase nuclease (Sigma) was added per sample. The samples were incubated at 37°C for 30 minutes. Samples were then sonicated to shear leftover DNA using Bioruptor Pico sonication system (Diogenode, sonication cycle: 30 sec ON/30 sec OFF, total sonication time: 5 cycles, temperature: 4°C). Protein concentration was determined by BCA assay (Thermo Fisher Scientific). 10–15 μg of protein lysates were run on 4–20% Criterion TGX Precast Midi Protein Gel (Bio-Rad) and then transferred to polyvinylidene difluoride membranes. Membranes were blocked in TBS with 0.1% Tween-20, 5% blotting grade blocker (Bio-Rad, 1706404XTU). The primary antibodies used are listed in Supplemental Table 1. Clarity or Clarity Max Western ECL Substrate (Bio-Rad 1705061, 1705062S) was used for imaging the blots. Blots were imaged on Bio-Rad ChemiDoc Touch Imaging System.

### Flow cytometry.

For B7H3 cell surface expression analysis, organoids were first dissociated into single cells using Accutase (STEMCELL Technologies, 07922). The resulting single cells were resuspended in PBS and stained with 1:1,000 Zombie Violet dye (Biolegend, 423113) for 15 minutes to exclude dead cells. Cells were pelleted and resuspended in 100 μl staining buffer (BioLegend). Fc receptors were blocked using Human TruStain FcX blocking solution (BioLegend) for 5 minutes at room temperature. Thereafter, cells were stained with human-EpCAM-APC (Miltenyi Biotec, 130-113-260) and CD276-PE (BioLegend, 331606) for 20 minutes at 4°C. The stained cells were washed, fixed in 4% paraformaldehyde for 10 minutes, and stored in staining buffer at 4°C up to 3–5 days. Flow cytometric analysis was done on BD LSR Fortessa cell analyzer using the BD FACSDiva software. Unstained cells and cells stained with isotype control-PE/APC antibodies were used for gating live cells and the false-positive peak, respectively. Median Fluorescent intensity(MFI) and percentage positive cells were analyzed using FlowJo software. Percentage positive cells were calculated by Overton cumulative histogram subtraction algorithm in Flowjo. Cell sorting was performed on FACSAria cell sorter (Becton Dickinson) using FACSDiva software.

### CRISPR/Cas9 experiments.

We used 3 organoid models for CRISPR experiments: LuCaP 145.2, LuCaP 173.1, and NCI-PC155. The 2 vector system (lentiCas9-Blast, lentiGuidePuro) was used. First, Cas9 expressing organoid lines were generated by lentiviral transduction of Cas9 plasmid (lentiCas9-Blast, addgene; 52962) and selection with Blasticidin for 10–14 days. Individual sgRNAs were cloned into lentiGuidePuro plasmid (NCI core facility). Cas9 expressing organoids were then transduced with lentiviral sgRNA (sgB7H3 no. 1; CAACCGCACGGCCCTCTTCCCGG) and selected with 1 μg/mL puromycin for 3 days. All lentiviral transductions were done by spin infection at 1,000*g*, for 90 minutes at 32°C.

Since clonal culture was not possible from organoids, B7H3 sgRNA–transduced LuCaP 145.2 organoids were processed into single cells and FACS sorted to enrich for B7H3-KO cells. For later experiments, FACS sorted B7H3-WT and B7H3-KO cells were maintained in 3D organoid cultures. B7H3 knockdown was periodically confirmed by Western blotting. Details of the CRISPR/Cas9 dropout screen are provided in supplemental methods.

### Preclinical trials.

Male NSG mice were obtained from the NCI Division of Cancer Treatment and Diagnosis (DCTD), Frederick and were s.c. injected with 2 million processed LuCaP PDX tumor cells or implanted with tumor pieces. When tumor exceeded approximately 150 mm^3^, mice were randomized into 3 groups: B7H3-PBD, R347-PBD, and vehicle. Then, 1 mg/kg of the B7H3-PBD or R347-PBD was administered i.p. weekly, for 2 weeks. Tumor growth (mm^3^) and body weight were measured twice a week. Tumor growth is shown for the individual mouse from the time of first treatment. Mice were euthanized if any of the 2 tumor diameters reached 2 cm or if their health was compromised.

For establishing LuCaP 136 metastasis model, processed PDX cells were first transduced with a luciferase reporter plasmid and reinjected s.c. in NSG mice. The resulting tumor was processed, and 100,000 single cells were injected intracardiacally. Tumor growth was measured by bioluminescence imaging. Treatment was started when the luminescence signal reached approximately 3 × 10^7^.

### ODXs.

ODXs were established in NOD scid γ (NSG) mice as previously described (21). The subsequent s.c. tumor was collected, processed, and 1–2 million cells, mixed with Matrigel in 1:1 ratio, were injected s.c. per mouse for the experiment. Mice were randomized into 3 treatment groups (B7H3-PBD, R347-PBD, and vehicle) when the tumors reached an average volume of 150–250 mm^3^. The treatment was started as described above.

### Toxicology studies with B7H3-PBD-ADC.

In addition to evaluating changes in body weight after administration of the ADC, we also performed full necropsy on day 8 and day 30 after treatment to evaluate acute and delayed toxicity, respectively. Tumor bearing mice (LuCaP 136 PDX model) were administered 2 i.p. doses of 1 mg/kg B7H3-PBD-ADC or control R347-PBD-ADC, given weekly for 2 weeks. A gross necropsy was performed, and a standard list of organs, including brain, lung, liver, kidney, spleen, thymus, testes, heart, and bone, were embedded in paraffin, sectioned, stained with H&E, and examined microscopically by a board-certified veterinary pathologist. Additionally, to assess accumulation of ADC in different organs, IHC for human IgG was performed on sections of liver, adrenal, kidney, and prostate collected on day 8 or day 30 after treatment from all animals. IHC assay positive control tumor tissue displayed appropriate IgG membrane immunoreactivity and no signal was observed in negative control tissues.

### RNA-Seq.

RNA-Seq was performed on the mPC organoid models on Illumina NovaSeq_S2 Flowcells using rRNA-depleted RNA and paired-end reads. Data were preprocessed and filtered using our previously described pipeline (21). RNA-Seq reads were aligned with STAR to the human genome, hg19. The featureCounts program from the subread package was applied to compute the raw reads count. All further analysis was performed in R. Data were TMM normalized to convert the raw read count to log_2_ transformed CPM (counts per million) using EdgeR. Median gene expression values were used for the biological replicates. Normalized log_2_CPM values were used for all subsequent analyses.

ConsensusDE package was used to perform differential expression analysis with 3 different algorithms (EdgeR, DESeq, and Voom) simultaneously. Genes were considered differentially expressed if adjusted Benjamin-Hochberg FDR was less than 0.05 for all 3 methods.

### Gene signature scores.

Single-sample gene set signature scores were calculated using GSVA with default parameters (33), using all MSigDB gene sets and IFN signature score (IFN score) refined for prostate cancer. Gene signature scores (IFN score, Repstress score, AR score, and RB score) used in this study are described in detail in the supplemental methods.

### Clinical data analyses.

SU2C/PCF 2019 data were downloaded from cbioportal (3). Log_2_(FPKM+1) mean–centered data was used. Since PolyA and capture sequenced RNA-Seq data are highly concordant (27), we merged the 2 data sets to have unique 328 samples. Samples with at least 30% tumor content (*n* = 303/328) were kept for analysis. Cbioportal data was merged with curations of the *TP53/RB1* status and AR/NE state from Nyquist et al. 2020 (27). IFN score and RB1 score were calculated using GSVA with method argument as “ssgsea” (33). For analysis of *SLFN11* status, data were normalized using ordered quantile normalization with the orderNorm function in R.

### Statistics.

R package, ggpubr, and GraphPad Prism were used for all analyses. Pearson’s correlation coefficient was calculated for scatterplots using ggscatter function in R. The nonparametric Kruskal Wallis test was used to compare multiple groups. For pairwise comparison between groups, Wilcoxon test was used with *P* value adjusted for multiple testing using the Holm method. Differences were considered significant for 2-tailed *P* values of < 0.05. For correlation analysis, Benjamini and Hochberg method was applied for multiple hypothesis test correction with adjusted *P* value (FDR) ≤ 0.05 indicating statistical significance. For the in vivo metastasis experiment, treatment response was compared using GraphPad Prism by 2-way Anova. Kaplan-Meier survival analysis was performed, and the survival rate was compared by Log-rank test. Data are expressed as the mean ± SEM, with *P* < 0.05 considered statistically significant. Heatmap was generated by using the R package ComplexHeatmap.

### Study approval.

Propagation of LuCaP PDX tumors and in vivo drug studies were performed at the NCI under NCI Animal Care and Use Committee–approved protocol. Tumors were periodically validated using STR analysis by Laragen Inc. All animal procedures were approved by NCI Animal Care and Use Committee. For organoids (NCI-PC44 and NCI-PC155) derived from patient biopsies, patients provided informed consent, and samples were obtained from the NIH clinical center under NIH IRB approval in accordance with U.S. Common Rule. All rapid autopsy tissues were collected from patients who had signed written informed consent under the aegis of the Prostate Cancer Donor Program at the University of Washington (2).

### Data and materials availability.

The sequencing data underlying this article have been deposited in Database of Genotypes and Phenotypes (dbGaP) and Gene Expression Omnibus (GEO) at https://www.ncbi.nlm.nih.gov/gap/ and https://www.ncbi.nlm.nih.gov/geo/, respectively, and can be accessed with phs001587.v2.p1 (dbGaP) and GSE236808 (GEO). All remaining data are available in the main text or the supplemental materials. Values for all data points in graphs are reported in the Supporting Data Values file.

## Author contributions

SA, EMH, and KK conceptualized the project. SA, LF, KM, JY, PSN, AM, CH, EMH, and KK developed the methodology. SA, LF, KM, JY, ANA, EC, MPR, LDT, RD, IC, JKL, MB, PSN, ISS, AM, and CH performed the experiments. SA, JB, ATK, BJC, AGS, ISS, PSN, AM, and CH were responsible for visualization. EMH and KK acquired funding. EMH and KK supervised the project. SA and KK wrote the original draft of the manuscript. SA, EMH, PSN, and KK were responsible for editing and review of the manuscript.

## Supplementary Material

Supplemental data

Supplemental data set 1

Supplemental data set 2

Supporting data values

## Figures and Tables

**Table 1:**
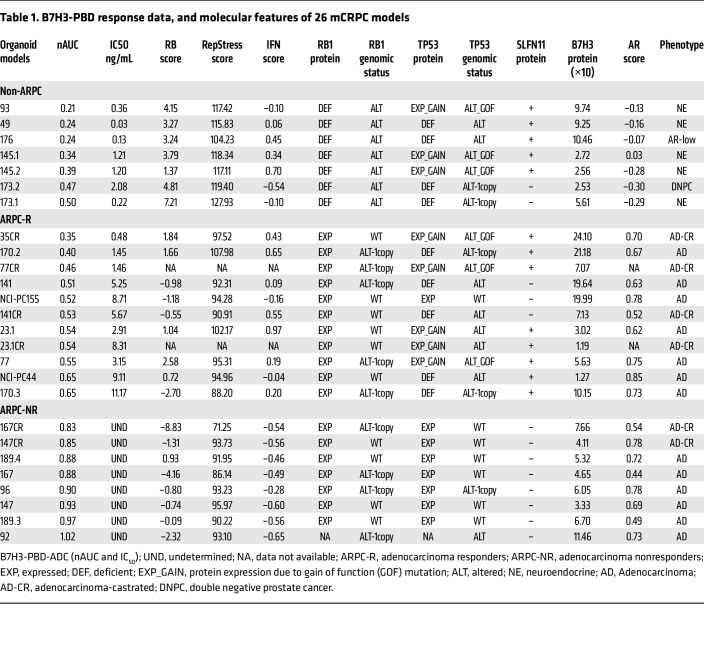
B7H3-PBD response data, and molecular features of 26 mCRPC models

## Version 1 09/19/2023

In-Press Preview

## Version 2 11/15/2023

Electronic publication
